# Expression of SNAIL in accompanying PanIN is a key prognostic indicator in pancreatic ductal adenocarcinomas

**DOI:** 10.1002/cam4.2016

**Published:** 2019-02-21

**Authors:** Sho Fujiwara, Yuriko Saiki, Kota Ishizawa, Shinichi Fukushige, Mie Yamanaka, Masaki Sato, Masaharu Ishida, Fuyuhiko Motoi, Michiaki Unno, Akira Horii

**Affiliations:** ^1^ Department of Molecular Pathology Tohoku University School of Medicine Sendai Japan; ^2^ Department of Surgery Tohoku University School of Medicine Sendai Japan

**Keywords:** epithelial‐to‐mesenchymal transition, high‐grade PanIN, pancreatic ductal adenocarcinoma, pancreatic intraepithelial neoplasia, prognosis

## Abstract

Pancreatic ductal adenocarcinoma (PDAC) is the most lethal cancer, mainly because of its invasive and metastatic characteristics. Pancreatic intraepithelial neoplasia (PanIN) is one of the major precursor lesions of PDAC. Although epithelial‐to‐mesenchymal transition (EMT) is known to play an important role for these malignant behaviors, the association between PanIN and EMT has not been clearly understood. Therefore, we explored possible molecules for regulation of EMT immunohistochemically. Using surgically resected specimens from 71 PDAC patients, expressions of SNAIL, SLUG, TWIST1, and ZEB1 were investigated in high‐grade PanIN (HG‐PanIN) and PDAC. Results demonstrated that PDAC accompanied by SNAIL‐positive HG‐PanIN showed a significantly better relapse‐free survival (RFS) (median survival time (MST) of 11.3 months vs 4.4 months, *P* < 0.001) and overall survival overall survival (OS) (MST of 25.2 months vs 13.6 months, *P* < 0.001). In PDAC accompanied by SLUG‐positive HG‐PanIN, RFS and OS (*P* = 0.09 and *P* = 0.05) tended to have a better prognosis. In contrast, we could not find any significant prognostic benefits in the expression of TWIST1 or ZEB1 in PDAC accompanied by HG‐PanIN. Our present results suggest that (1) EMT may play an important role in the development of PDAC from HG‐PanIN, and (2) SNAIL may predict a distinct subgroup that shows a better prognosis.

## 1 INTRODUCTION

Pancreatic ductal adenocarcinoma (PDAC) is the most lethal cancer in the United States and Japan, because early detection of PDAC is very difficult, and in many cases, patients are diagnosed at an advanced stage with metastasized or invaded disease.^1^, ^2^

Numerous studies have indicated an association between epithelial‐to‐mesenchymal transition (EMT) and the poor prognosis of PDAC.^3^, ^4^ EMT is known to be one of the key factors in the progression to invasion, metastasis, and dissemination of PDAC.^5^ Furthermore, EMT plays an important role in the acquisition of chemoresistance in pancreatic cancer.^6^ In particular, the major EMT regulators, SNAIL, SLUG, TWIST1, and ZEB1, contribute not only to invasion and metastasis but also to stemness and apoptosis; these are the most widely investigated EMT‐inducing molecules in various cancers.^7^, ^8^, ^9^, ^10^, ^11^

Recent studies have demonstrated that pancreatic intraepithelial neoplasia (PanIN) is one of the major precursor lesions of PDAC.^12^, ^13^, ^14^ PanIN lesions are thought to develop by stepwise accumulation of genetic and epigenetic alternations in a progression from low‐grade PanIN (LG‐PanIN) to high‐grade PanIN (HG‐PanIN) and finally to PDAC.^12^, ^13^, ^15^ PanIN exhibits different types of mutations than intraductal papillary mucinous neoplasm (IPMN) as tumorigenesis develops and progresses.^16^ Although HG‐PanIN is an important precursor lesion of PDAC, it is rarely diagnosed; most HG‐PanIN lesions are found incidentally in resected specimens that include PDAC.^15^, ^16^ Therefore, the expression of regulators of EMT in HG‐PanIN remains unclear. In addition, the details regarding the mechanisms of PDAC development from PanIN lesions are also largely unknown. The present study aimed to explore the functions of SNAIL, SLUG, TWIST1, and ZEB1, regulators of EMT for possible effects in prognosis of patients with PDAC that are accompanied by HG‐PanIN.

## 2 MATERIALS AND METHODS

### 2.1 Patients and specimens

The surgical specimens resected from patients with PDAC between 2003 and 2010 in Tohoku University Hospital. These specimens are the only tissues we have that allow us to follow prognoses and use for immunohistochemical analyses. Ethical approval was obtained from the Ethics Committee of the Tohoku University School of Medicine under the accession numbers of 2015‐1‐473 and 2015‐1‐474. From the 73 specimens, we identified 32 PanIN cases. Among these, 27 PanIN3 specimens were classified as HG‐PanIN (summarized in Table 1). Specimens of HG‐PanIN were diagnosed at the Department of Pathology, Tohoku University Hospital, and redetermined by a Certified Pathologist of The Japanese Society of Pathology (YS).

**Table 1:** HG‐PanIN patients’ characteristics

Age	
Average	71
Range	58‐82
Gender	
Male	14
Female	13
Operation	
Pancreaticoduodenectomy	15
Distal pancreatectomy	8
Total pancreatectomy	4
UICC stage	
IB	1
IIA	4
IIB	9
IV	13
UICC T	
1	0
2	1
3	25
4	1
UICC N	
0	5
1	22
UICC M	
0	18
1	9
ly	
0	1
1	2
2	16
3	8
v	
0	1
1	5
2	15
3	6
ne	
0	1
1	3
2	4
3	19

### 2.2 Immunohistochemistry

Immunohistochemical staining was performed on 4‐μm‐thick serial sections from formalin‐fixed paraffin‐embedded resected specimens, and antibodies against SNAIL (ab180714, Abcam), SLUG (ab128485, Abcam or LS‐C175177‐100, LifeSpan Bio), TWIST1 (ab50581, Abcam), and ZEB1 (NBP1‐05987, Novus) were used. Heat‐mediated antigen retrieval was performed using 0.1 mol L^−1^ citrate buffer for 30 minutes by microwave for SNAIL and SLUG, and using 1 mmol L^−1^ EDTA + 10 mmol L^−1^ Tris for 30 minutes by microwave for TWIST1 and ZEB1. Endogenous peroxidase activity was blocked with incubation in 1% hydrogen peroxidase in methanol for 15 minutes. Primary antibodies were treated with PBS overnight at 4°C at the following dilutions: 1:400 (SNAIL), 1:150 (SLUG), 1:400 (TWIST1), and 1:200 (ZEB1); secondary antibodies were treated according to the manufacturers’ recommendations. Immunohistochemical staining was performed using 3,3′‐diaminobenzidine for the following durations: 5 minutes for SNAIL, 10 minutes for SLUG and TWIST1, and 15 minutes for ZEB1. All slides were counterstained with hematoxylin, dehydrated in alcohol, and covered for analysis.

### 2.3 Definitions and evaluations of immunohistochemistry

**Figure 1:**
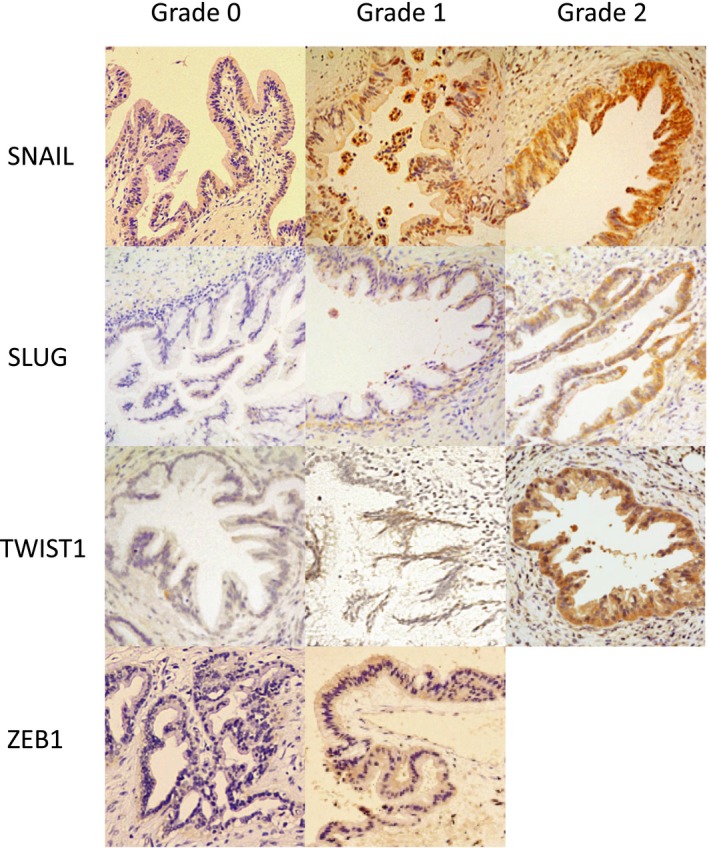
Immunohistochemical staining results of EMT regulators in HG‐PanIN: SNAIL, SLUG, TWIST1, and ZEB1. We categorized and defined three grades: grade 0, <10% positive staining; grade 1, 10%‐50% positive; grade 2, 50%< positive

Based on the immunostainings, we categorized and sorted the specimens into the following three groups; grade 0, 0%‐10%; grade 1, 10%‐50%; and grade 2, 50% or more cells were positively stained (Figure 1). These specimens were reviewed by two pathologists independently using our criteria.

### 2.4 Statistical analysis

We performed the paired *t* test to evaluate the associations of EMT regulator status in HG‐PanIN lesions and PDAC. To analyze the correlations of EMT status with the clinicopathological features of HG‐PanIN and PDAC statistically, Fisher's exact test or the Chi‐squared test was used. Overall survival rate (OS) and relapse‐free survival rate (RFS) were evaluated using the Kaplan‐Meier method and analyzed using the log‐rank test in JMP Pro version 14 (JMP, SAS Institute). Significant association was defined as a *P* value below 0.05.

## 3 RESULTS

### 3.1 Expressions of possible EMT regulators

**Figure 2:**
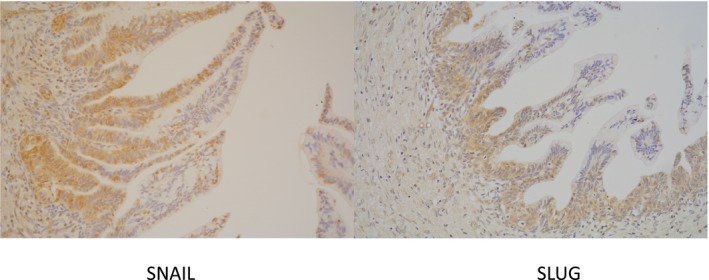
Expression of SNAIL and SLUG in HG‐PanIN and PDAC. Expression levels in PDAC regions were higher than those in HG‐PanIN, and expressions in invading PDAC regions are prominent

SNAIL, SLUG, and TWIST1 were identified in both the cytoplasm and nuclei of HG‐PanIN and PDAC cells, although they were more concentrated in the nuclei. ZEB1 was identified in a few nuclei in HG‐PanIN and PDAC cells, but was negative in most of them. The nuclei of fibroblasts in the mesenchyme surrounding PDAC were positive for ZEB1. Representative results of immunohistochemical staining are illustrated in Figure 2.

### 3.2 Correlations between possible EMT regulators in HG‐PanIN and PDAC

**Figure 3:**
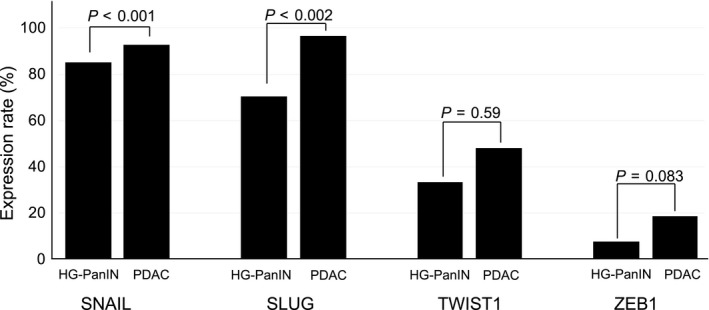
Expression levels of EMT regulators and relationships between HG‐PanIN and PDAC. All regulators’ expressions increased in PDAC compared with HG‐PanIN. There were statistically significant differences in SNAIL and SLUG, respectively

Positive staining (grades 1 and 2) for SNAIL and SLUG were observed in 70% to 95% of HG‐PanIN and PDAC cells (Figure 3 and Table 2). However, TWIST1 expression was low; in HG‐PanIN, 33.3% were positive (grade 1, 14.8% and grade 2, 18.5%) and, in PDAC, 48.1% (grade 1, 37.0% and grade 2, 11.1%) were positive. Furthermore, in both HG‐PanIN and PDAC, ZEB1 was less expressed than the other regulators; 7.4% and 18.5%, respectively, were positive (Figure 3 and Table 2). No grade 2 (positive staining of 50% or more) specimens were observed. PDAC showed more frequent expression than HG‐PanIN for all four molecules (Figure 3).

**Table 2:** Expression levels of EMT regulators in HG‐PanIN and PDAC lesions

	HG‐PanIN			PDAC		
	Grade 0	Grade 1	Grade 2	Grade 0	Grade 1	Grade 2
	<10%	10‐50%	50%<	<10%	10‐50%	50%<
SNAIL	4/27 (14.8%)	11/27 (40.7%)	12/27 (44.4%)	2/27 (7.4%)	3/27 (11.1%)	22/27 (81.5%)
SLUG	8/27 (29.6%)	9/27 (33.3%)	10/27 (37.0%)	1/27 (3.7%)	10/27 (37.0%)	16/27 (59.3%)
TWIST1	18/27 (66.7%)	4/27 (14.8%)	5/27 (18.5%)	14/27 (51.9%)	10/27 (37.0%)	3/27 (11.1%)
ZEB1	25/27 (92.6%)	2/27 (7.4%)	0/27 (0.0%)	22/27 (81.5%)	5/27 (18.5%)	0/27 (0.0%)

Paired *t* tests to determine the possible association between EMT regulators and tumorigenesis revealed that expressions of SNAIL and SLUG significantly increased in PDAC compared with HG‐PanIN (*P* < 0.001 and <0.002). Although TWIST1 and ZEB1 were more expressed in PDAC than in HG‐PanIN, the differences were not statistically significant (Figure 3).

### 3.3 Correlation between possible EMT regulators in HG‐PanIN and patient prognosis

We investigated possible correlations between the expression of EMT regulators in HG‐PanINs and the clinicopathological features of PDAC. Results are summarized in Table 3. There were no significant differences in any of the following factors; patient's age, gender, UICC Stage, UICC T factors, UICC N factors, UICC M factors, vascular invasion, lymphatic invasion, neural invasion, or surgical resectability status.

**Table 3:** EMT status in HG‐PanIN and clinicopathological features. There were any statistically significant differences between EMT positive groups and negative groups

		SNAIL			SLUG			TWIST1			ZEB1		
		Negative (n = 4)	Positive (n = 23)	*P* value	Negative (n = 8)	Positive (n = 19)	*P* value	Negative (n = 18)	Positive (n = 9)	*P* value	Negative (n = 25)	Positive (n = 2)	*P* value
Age													0.96
70		2 (50.0)	11 (47.8)	0.94	4 (50.0)	9 (47.4)	0.9	9 (50.0)	4 (44.4)	0.79	12 (52.0)	1 (50.0)	
71		2 (50.0)	12 (52.2)		4 (50.0)	10 (52.6)		9 (50.0)	5 (44.4)		13 (52.0)	1 (50.0)	
Gender													
Female		1 (25.0)	12 (52.2)	0.32	4 (50.0)	9 (47.4)	0.9	10 (55.6)	3 (33.3)	0.28	13 (52.0)	0 (0.0)	0.16
Male		3 (75.0)	11 (47.8)		4 (50.0)	10 (52.6)		8 (44.4)	6 (66.7)		12 (48.0)	2 (100.0)	
UICC T													
T1,2		0 (0.0)	1 (4.3)	0.57	1 (12.5)	0 (0.0)	0.12	1 (5.6)	0 (0.0)	0.36	1 (4.0)	0 (0.0)	0.69
T3,4		4 (100.0)	22 (95.7)		7 (87.5)	19 (100.0)		17 (94.4)	9 (100.0)		24 (96.0)	2 (100.0)	
UICC N													
N0		1 (25.0)	4 (17.4)	0.73	1 (12.5)	4 (21.0)	0.59	4 (22.2)	1 (11.1)	0.47	5 (20.0)	0 (0.0)	0.36
N1		3 (75.0)	19 (82.6)		7 (87.5)	15 (79.0)		14 (77.8)	8 (88.9)		20 (80.0)	2 (100.0)	
UICC M													
M0		3 (75.0)	15 (65.2)	0.7	4 (50.0)	14 (73.7)	0.23	12 (66.7)	6 (66.7)	1	17 (68.0)	1 (50.0)	0.61
M1		1 (25.0)	8 (24.8)		4 (50.0)	5 (26.3)		6 (33.3)	3 (33.3)		8 (32.0)	1 (50.0)	
Vascular invasion													
v0,1		0 (0.0)	3 (13.0)	0.31	1 (12.5)	2 (10.5)	0.88	3 (16.7)	0 (0.0)	0.12	3 (12.0)	0 (0.0)	0.48
v2,3		4 (100.0)	20 (87.0)		7 (87.5)	17 (89.5)		15 (83.3)	9 (100.0)		22 (88.0)	2 (100.0)	
Lymphatic invasion													
ly0.1		1 (25.0)	5 (21.7)	0.89	1 (12.5)	5 (26.3)	0.41	5 (27.8)	1 (11.1)	0.3	5 (20.0)	1 (50.0)	0.37
ly2,3		3 (75.0)	18 (78.3)		7 (87.5)	14 (73.7)		13 (72.2)	8 (88.9)		20 (80.0)	1 (50.0)	
Neural invasion													
ne0,1		0 (0.0)	4 (17.4)	0.37	2 (25.0)	2 (10.5)	0.35	4 (22.2)	0 (0.0)	0.058	3 (12.0)	1 (50.0)	0.22
ne2,3		4 (100.0)	19 (82.6)		6 (75.0)	17 (89.5)		14 (77.8)	9 (100.0)		22 (88.0)	1 (50.0)	
Surgical resectability status													
R0		4 (100.0)	19 (82.6)	0.24	8 (100.0)	15 (79.0)	0.16	16 (88.9)	7 (77.8)	0.44	21 (84.0)	2 (100.0)	0.41
R1		0 (0.0)	4 (17.4)		0 (0.0)	4 (21.0)		2 (11.1)	2 (22.2)		4 (16.0)	0 (0.0)	

**Figure 4:**
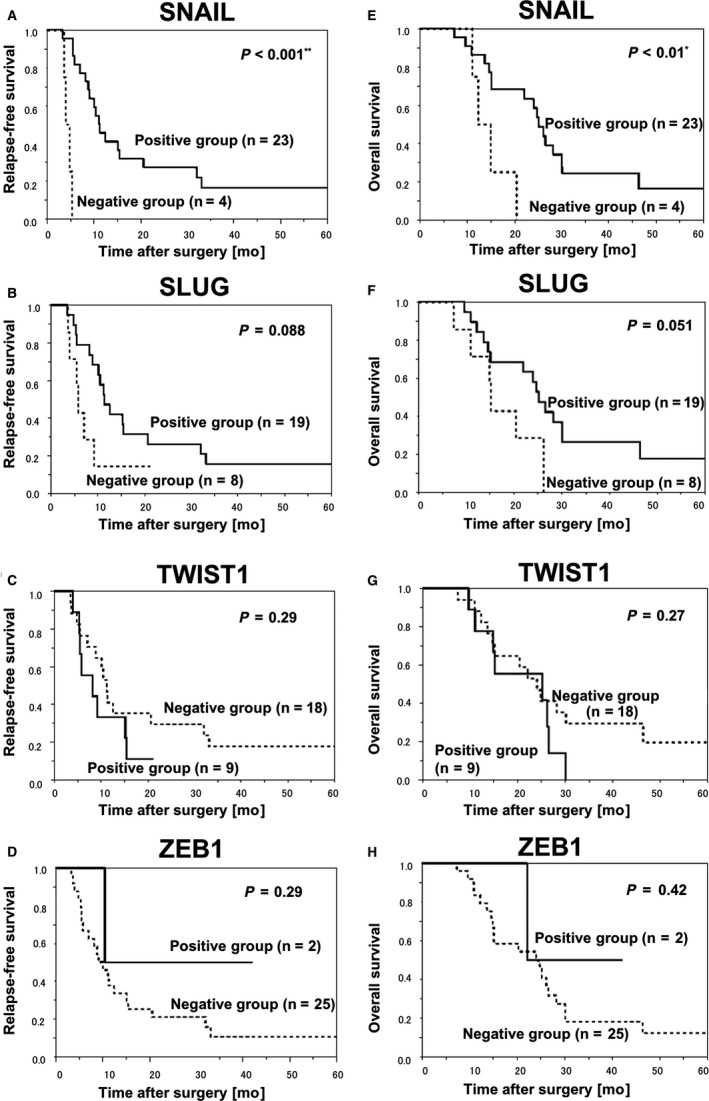
Kaplan‐Meier survival curves by expression status of each EMT regulator in accompanying HG‐PDAC and patients’ prognoses. (A‐D) RFS; and (E‐H) OS. SNAIL (A and E), SLUG (B and F), TWIST1 (C and G), ZEB1 (D and H)

We also investigated the possible correlations between EMT regulators and prognosis (Figure 4). The SNAIL‐positive group had a significantly good prognosis; RFS (log‐rank, median survival time (MST) 11.3 months vs 4.4 months, *P* < 0.001) and OS (log‐rank, MST 25.2 months vs 13.6 months, *P* < 0.001). The SLUG‐positive group tended to have a good prognosis, although the prolongation was not statistically significant; RFS (log‐rank, MST 11.4 months vs 5.8 months, *P* = 0.09) and OS (log‐rank, MST 24.1 months vs 15.2 months, *P* = 0.05). Neither TWIST1 nor ZEB1 showed any significant association (Figure 4).

### 3.4 Correlation between SNAIL expression in PDAC with and without accompanying HG‐PanIN and prognosis

As mentioned above, there was a significant association between SNAIL‐positive HG‐PanIN and better prognosis, so we further analyzed PDAC group and found that SNAIL‐positive patients also showed better RFS (log‐rank, MST 10.8 months vs 4.4 months, *P* < 0.001) and OS (log‐rank, MST 24.8 months vs 13.6 months, *P* = 0.048) when compared with SNAIL‐negative PDAC group (Figure S1). On the other hand, patients with PDAC whose tumors were not accompanied by HG‐PanIN did not show any differences (Figure S1).

In all 73 PDAC cases, we also studied the expressions of SNAIL, SLUG, TWIST1, and ZEB1 immunohistochemically, irrespective of co‐existence of PanIN, and successfully analyzed 71 cases; no significant associations between RFS and OS were observed (S. Fujiwara, Y. Saiki, K. Ishizawa, S. Fukushige, M. Yamanaka, M. Sato, M. Ishida, F. Motoi, M. Unno, A. Horii, submitted).

## 4 DISCUSSION

In this study, we investigated HG‐PanIN and PDAC tissues and immunohistochemically analyzed the expression of four major EMT regulators: SNAIL, SLUG, TWIST1, and ZEB1. Our results suggest that positive expression of SNAIL in HG‐PanIN was a statistically positive prognostic factor for RFS and OS. Positive SLUG in HG‐PanIN also tended to indicate a better prognosis, although the association was not statistically significant. This study investigated a small number of samples; more case accumulation might clarify whether SLUG is a prognostic factor for OS. On the other hand, neither TWIST1 nor ZEB1 had any significant associations with HG‐PanIN.

Previous studies have shown that expressions of EMT regulators are poor prognostic factors for tumorigenesis and are positively associated with invasions and metastasis.^3^, ^4^ A retrospective study reported that high expression of ZEB1 in PDAC indicated a poorer prognosis; MST was 10.2 months vs 17.1 months (*P* = 0.002) in disease‐free survival and 17.0 months vs 24.4 months in OS (*P* = 0.057).^3^ Another study revealed that malignant IPMN tumors express high ZEB1.^4^ In our results, PDAC patients accompanied by SNAIL‐expressing HG‐PanIN did have better prognoses than those with negative expressions, and PDAC patients with SLUG‐expressing HG‐PanIN tended to have better prognoses; these are paradoxical results because EMT is thought to associate with invasion and metastases as well as chemoresistance.^3^, ^4^, ^17^ Some previous studies have reported that PDAC patients had good prognoses if PanIN is present.^16^ Moreover, the presence of HG‐PanIN in PDAC is associated with better OS than low‐grade PanIN or non‐PanIN in PDAC.^16^ Another similar study revealed that the participant group without PanIN in resected PDAC tended to have a poorer survival after resection than the group with PanIN.^18^ However, these studies did not analyze the EMT‐inducing molecules in PanIN. Our present study highlighted SNAIL as one of the key players in the regulating process of patients’ prognoses, and it seems possible that some gene(s) or molecule(s) downstream of SNAIL may play crucial role(s) that leads to better prognoses for PDAC patients. If this is the case, then such gene(s) or molecule(s) should be associated with modest invasion and/or growth, and other PDAC cells grow or invade so rapidly that accompanying HG‐PanIN is rarely found.

We verified the association between EMT and PanIN, suggesting HG‐PanIN may be classified into subgroups depending on EMT status. PanIN is a heterogenous lesion. It is known that accumulations of genetic and/or epigenetic alterations are necessary to progress to PDAC. EMT is a very complex phenomenon, but our results suggest that EMT plays a role in the tumorigenesis of some HG‐PanIN and may be a distinct subtype of PDAC.

There are some limitations to our study. First, it is a retrospective study with a relatively small sample size. Second, although PanIN and PDAC are known to be genetically and/or epigenetically heterogenous, we classified the total score of the levels of expression of EMT regulators in each patient. In future studies, more cases should be accumulated, and more detailed genetic analyses should be performed, considering the heterogeneity of each tumor cell type. We may also need to consider a control mechanism involving the immunological systems as another factor, resulting in favorable prognoses for the EMT positive group.

In conclusion, we proposed that some EMT regulators, SNAIL and possibly SLUG, lead to a distinct subgroup of PDAC with favorable prognoses. These results indicate possible new targets for treatments and early detection markers.

## CONFLICT OF INTEREST

The authors declare no conflicts of interest.
