# Supplementary material for: Expression of SNAIL in accompanying PanIN is a key prognostic indicator in pancreatic ductal adenocarcinomas
Source: Cancer Med. 2019 Feb 21;8(4):1671–8. doi: 10.1002/cam4.2016 (PMC6488215; doi:10.1002/cam4.2016)
Supplement: Supplementary file 1 [file CAM4-8-1671-s001.pdf]

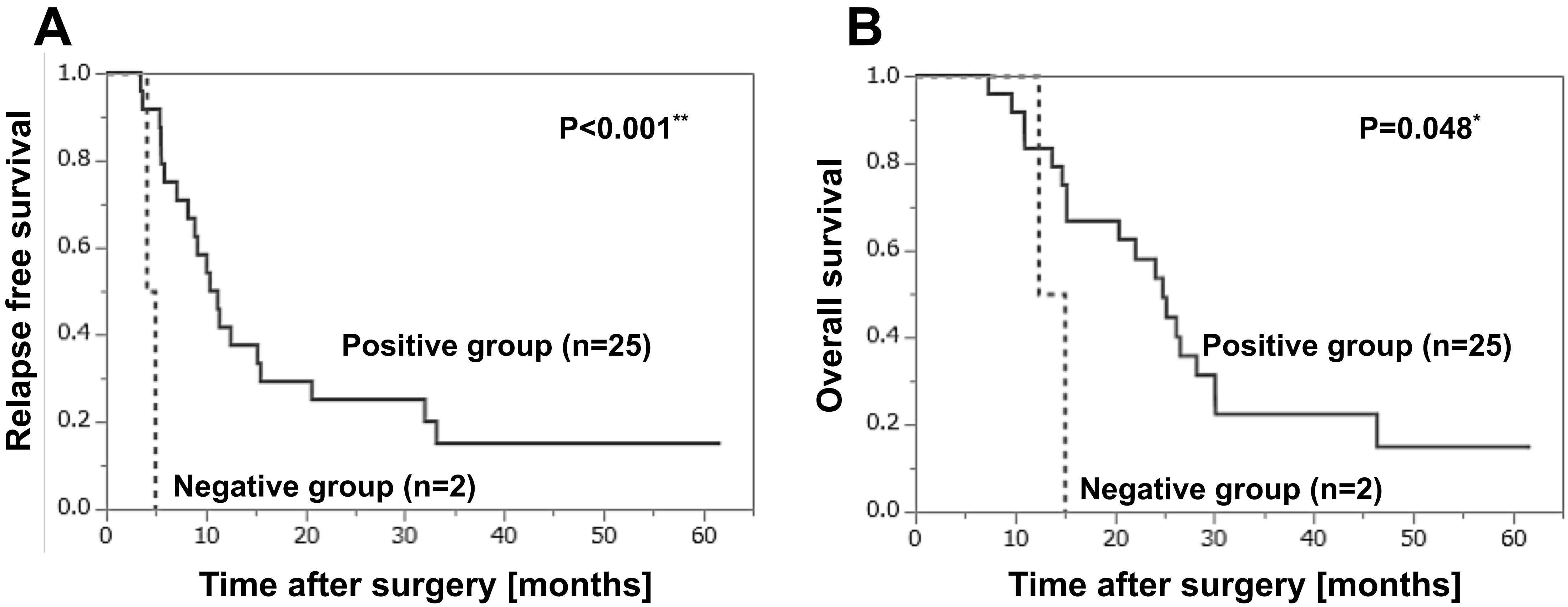

Kaplan–Meier survival curves by expression status of SNAIL in PDAC and patients' prognoses. A and B, results of RFS and OS, respectively.
